# Beyond the target: new dimensions of antifungal action

**DOI:** 10.1038/s41420-026-03266-w

**Published:** 2026-07-21

**Authors:** Marina Campos Rocha, Neta Shlezinger, Jean-Pierre Gangneux, Nicolas Papon

**Affiliations:** 1 Koret School of Veterinary Medicine, Faculty of Agriculture, The Hebrew University, Rehovot, Israel; 2 University of Rennes, CHU Rennes, Inserm, EHESP, Irset (Institut de Recherche en Santé, Environnement et Travail) UMR_S, Rennes, France; 3 Laboratory of Parasitology and Medical Mycology, European Confederation of Medical Mycology (ECMM) Excellence Center, Centre National de Référence Aspergilloses Chroniques, Rennes University Hospital, Rennes, France; 4 Univ Angers, Univ Brest, IRF, SFR ICAT, Angers, France

**Keywords:** Fungal infection, Target identification

Fungal diseases remain a major but underestimated cause of morbidity and mortality, with severe infections now estimated to contribute to millions of deaths annually [1]. Despite this burden, available antifungal options remain limited. Systemic antifungal therapy still relies mainly on three major classes: polyenes, azoles, and echinocandins [2]. Echinocandins are recommended as first-line therapy for many types of invasive candidiasis, particularly candidemia in hospitalized patients [3]. Their clinical value comes from safety, potent activity against *Candida*, and a unique mechanism: non-competitive inhibition of (1,3)-β-glucan synthase (encoded by the *FKS* gene), a fungal enzyme absent in mammals.

The classical view of echinocandins as highly specific (1,3)-β-glucan synthase inhibitors is now being refined. Resistance is an increasing clinical problem, especially with *Candida auris* and *Nakaseomyces glabrata*, identified as emerging multidrug-resistant pathogens listed by the World Health Organization as a critical-priority fungal pathogens in 2022 [4, 5]. Mutations in *FKS1* and *FKS2*, which encode (1,3)-β-glucan synthase in *Candida* spp., remain the major recognized mechanism of resistance to echinocandins [6]. Yet wild-type *FKS* isolates can exhibit reduced susceptibility, implying additional regulatory layers. In this respect, an emerging factor is the membrane environment where β-glucan synthase functions. Recent structural studies show (1,3)-β-glucan synthase has a crucial membrane-embedded structure, including transmembrane regions linked to echinocandin resistance [7]. In parallel, local lipid organization, including sterol-rich environments, can shape enzyme behavior and influence drug susceptibility [8]. Thus, the fungal plasma membrane is not merely a passive scaffold for the drug target but a potential modulator of echinocandin action, suggesting that it contributes to echinocandin activity.

**Fig. 1:**
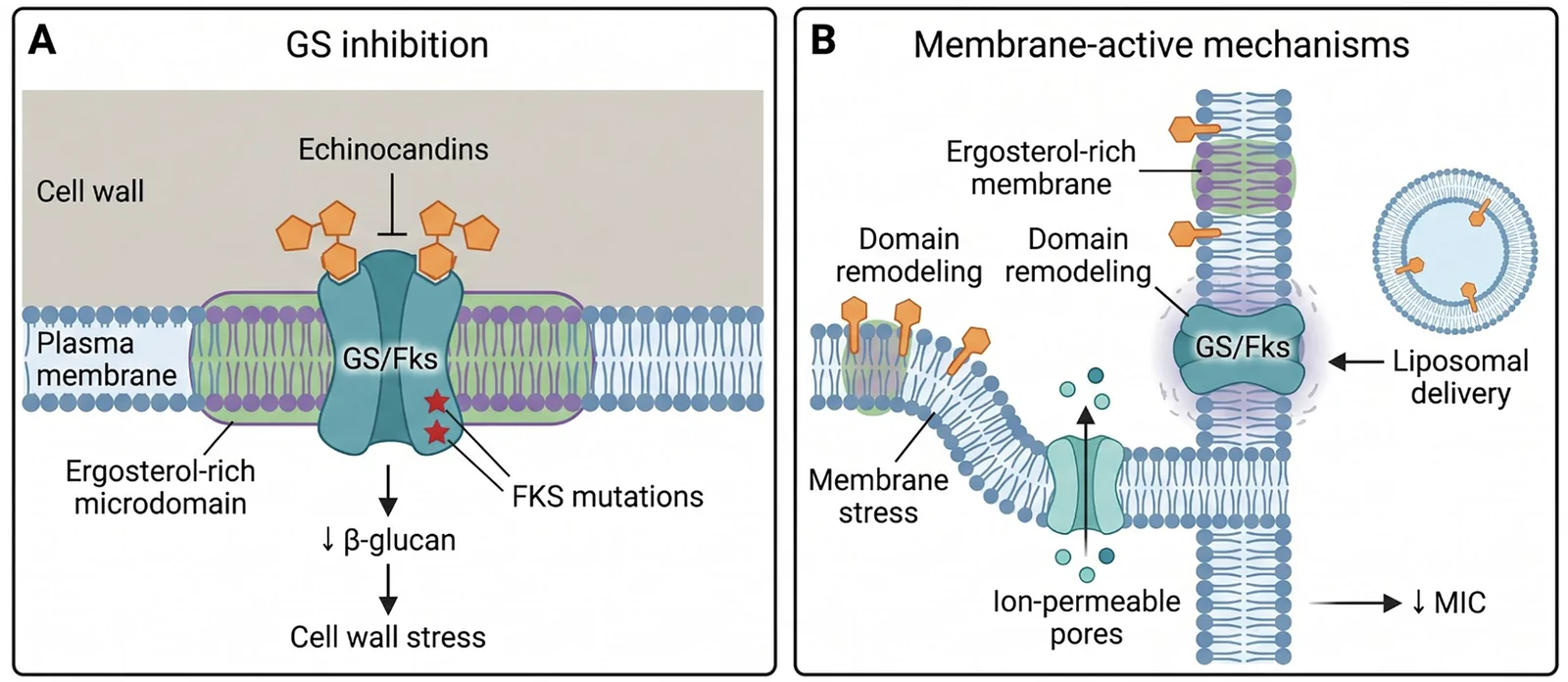
The target–membrane–formulation model of echinocandin action. **A** In the classical model, echinocandins inhibit (1,3)-β-glucan synthase (GS/Fks), reducing β-glucan synthesis and weakening the fungal cell wall. Resistance is primarily associated with hotspot mutations in *FKS1/FKS2* gene sequences that reduce echinocandin susceptibility. **B** Malykhina et al. expand this model by showing that echinocandins also interact directly with ergosterol-rich biomimetic membranes, promoting lipid-domain remodeling, membrane stress, and ion-permeable pore formation in simplified lipid systems. Liposomal delivery may further enhance drug access to fungal membrane environments and reduce MICs in clinical *Candida* isolates in vitro. Together, these findings suggest that echinocandin efficacy may be shaped by the enzyme target, the sterol-rich membrane context, and formulation-dependent delivery.

Malykhina and colleagues now provide direct experimental support for this idea in biomimetic membrane systems, showing that echinocandin drugs anidulafungin, caspofungin, and micafungin interact with model lipid membranes in a sterol-dependent manner, independent of (1,3)-β-glucan synthase. The importance of the study is not simply that echinocandins perturb membranes, but that it reframes their activity as a property of three linked variables: the (1,3)-β-glucan synthase target, the sterol-rich membrane environment, and formulation-dependent drug access. This target–membrane–formulation model provides a more expansive framework for understanding echinocandin activity, resistance, and future drug optimization [8] (Fig. 1).

In a first series of experiments, Malykhina et al. combined complementary membrane-based approaches to test whether echinocandins can act directly on fungal biomembranes. Across defined biomimetic lipid systems, including ergosterol and cholesterol-containing membranes, they used diverse approaches to determine how anidulafungin, caspofungin, and micafungin interact with lipid bilayers. Differential scanning calorimetry, molecular dynamics simulations, and calcein-leakage assays all point in the same direction: anidulafungin, caspofungin, and micafungin interact more strongly with ergosterol-containing bilayers than with cholesterol-containing bilayers. These experiments do not establish how membrane perturbation contributes to antifungal killing in living cells, but they provide a mechanistic basis for why fungal-like membranes may favor echinocandin activity. This is consistent with a prior cell biology study showing that lipid composition modulates echinocandin susceptibility. In *Nakaseomyces glabrata*, altered sphingolipid biosynthesis and reduced ergosterol ordering have been linked to resistance [7], whereas disruption of membrane lipid asymmetry and integrity can sensitize *Cryptococcus neoformans* to caspofungin [9, 10].

Then, Malykhina et al. also provide direct evidence that echinocandins influence lateral membrane organization in simplified lipid systems. The drugs increased line tension at liquid-ordered/liquid-disordered boundaries and promoted the coalescence of ordered lipid domains. This observation is relevant because (1,3)-β-glucan synthase is not randomly distributed in the fungal plasma membrane. Indeed, the enzyme participates in higher-order plasma membrane organization shaped by the local lipid environment [7]. If similar membrane remodeling occurs in fungal cells, echinocandins could influence (1,3)-β-glucan synthase activity not only by binding the enzyme, but also by altering the local lipid environment required for optimal enzyme activity. At present, this remains an extrapolation from simplified membrane systems rather than a demonstrated cellular mechanism.

Another key finding of this enlightening report is that all three echinocandins can form small ion-permeable pores in ergosterol-rich planar lipid bilayers, with distinct conductance-selectivity profiles, consistent with their structural differences. Molecular dynamics simulations further suggested that echinocandin-induced lipid disordering reduces membrane bending rigidity, thereby lowering the energetic barrier to pore formation, compared with pores formed by amphotericin B [11]. This does not prove that pore formation is a primary killing mechanism but establishes it as a plausible contributor to membrane stress, ion imbalance, or drug potentiation under specific cellular conditions.

Overall, the study’s central advance is repositioning the fungal membrane as an active pharmacological variable. In the classical model, echinocandin activity is explained by drug binding to (1,3)-β-glucan synthase and by resistance mutations in *FKS* genes. Malykhina et al. do not overturn this model, but they make it incomplete. Their data suggest that the membrane surrounding (1,3)-β-glucan synthase may influence drug behavior, local enzyme organization, and possibly the downstream cellular consequences of echinocandin exposure. At present, this remains a biophysical and mechanistic framework rather than a fully demonstrated cellular mechanism. The next critical step will be to determine whether membrane perturbation, domain remodeling, or pore formation measurably contributes to echinocandin activity in fungal cells and during infection.

These findings also have implications for how echinocandin resistance is interpreted. Resistance surveillance focuses largely on mutations in *FKS1* and *FKS2*, the best-established genetic markers of reduced echinocandin susceptibility [12]. The Malykhina study does not demonstrate that membrane remodeling is a clinical resistance mechanism, but it raises a framework for investigating non-FKS contributions to reduced antifungal susceptibility. If membrane-directed activity contributes to drug efficacy in living cells, however, *FKS* genotype alone may not capture the full resistance phenotype. Isolates with wild-type *FKS* alleles but altered membrane composition or organization could exhibit reduced susceptibility through mechanisms not detected by standard genotyping. Conversely, isolates carrying *FKS* mutations may differ in residual drug vulnerability depending on the permissiveness of their membrane environment. This framework could help explain one layer of the imperfect alignment among MIC values, *FKS* genotype, and clinical outcome, although that discordance also reflects host status, drug exposure, and infection site. The membrane component will therefore require validation in panels of clinical isolates and infection models before it can be considered a clinically relevant resistance mechanism.

The liposomal delivery experiments raise a second, more translational possibility: formulation may modulate the membrane-directed component of echinocandin activity. Liposomal amphotericin B provides a precedent for how reformulation can reshape the therapeutic behavior of a membrane-active antifungal, although the clinical rationale for amphotericin B liposomes is not directly transferable to echinocandins [11]. For echinocandins, the key question is different: can formulation improve their effective access to fungal membranes or membrane domains, thereby enhancing activity against isolates with reduced susceptibility? The current data support formulation as an experimental direction, not yet as a clinical strategy. Pharmacokinetics, tissue distribution, fungal cell targeting, toxicity, formulation stability, and efficacy in infection models will all need to be evaluated.

More broadly, the study illustrates why established antifungal drugs should be revisited not only as target-directed inhibitors but also as molecules whose activities are shaped by complex, specific cellular architectures. Mandimycin is a recent example of the same idea: changing glycosylation within a polyene scaffold can shift membrane recognition away from the ergosterol-centered mode of amphotericin B and toward fungal phospholipids [13], suggesting that sterol dependence, lipid selectivity, and membrane organization may serve as pharmacological variables. In the case of azoles, the implications are distinct: by disrupting ergosterol biosynthesis, azoles induce membrane states that can either heighten susceptibility to additional stress or diminish the effectiveness of drugs that depend on ergosterol-rich environments. Consequently, combinations involving echinocandins, azoles, and polyenes should be evaluated in relation to both their molecular targets and the membrane states generated by each drug. This may also be relevant to newer agents targeting the (1,3)-β-glucan synthesis pathway. Rezafungin [14], a next-generation echinocandin, is a potential candidate for similar membrane-based analyses. Ibrexafungerp is also an interesting candidate, although it is not an echinocandin; it is a triterpenoid (1,3)-β-glucan synthase inhibitor active against some *Candida* isolates with reduced echinocandin susceptibility [15]. For echinocandins, the relevant pharmacological determinant of activity may not be the drug and (1,3)-β-glucan synthase alone, but the drug, the enzyme, the sterol-rich membrane environment, and the formulation that governs drug access to that environment.

This expands the model of echinocandin action from a single-target mechanism to a membrane-contextualized pharmacological process. The same framework may help reassess echinocandin-based drug combinations, in which effects on sterol metabolism, membrane organization, or cell wall stress could influence whether the combinations become synergistic or antagonistic. It also gives genetic lipid remodeling a clearer place in resistance biology. Modifications in sterol regulation, sphingolipid metabolism, or lipid-flippase activities can impact membrane architecture, lipid asymmetry, and the microenvironment surrounding membrane-associated targets [7, 9, 10, 16], thereby contributing to adaptive reductions in susceptibility, particularly in isolates where the FKS genotype does not fully explain the observed phenotype. The next challenge is to determine whether this membrane-active behavior remains pharmacologically meaningful in fungal cells, clinical isolate panels, and infection systems, where its contribution to resistance, efficacy, and therapeutic optimization can be directly tested.
